# Development of IoT-Based Particulate Matter Monitoring System for Construction Sites

**DOI:** 10.3390/ijerph182111510

**Published:** 2021-11-01

**Authors:** Hyunsik Kim, Sungho Tae, Pengfei Zheng, Geonuk Kang, Hanseung Lee

**Affiliations:** 1 Department of Architectural Engineering, Hanyang University, Ansan 15588, Korea; visionysj@gmail.com (H.K.); zpf970802@hanyang.ac.kr (P.Z.); 2 School of Architecture and Architectural Engineering, Hanyang University, Ansan 15588, Korea; ercleehs@hanyang.ac.kr; 3 Daedan Inc., 155, Ansandaehak-ro, Ansan 15328, Korea; daedan79@gmail.com

**Keywords:** construction site, dust, particulate matter, IoT, monitor

## Abstract

Particulate matters (PMs) generated on construction sites can pose serious health risks to field workers and residents living near construction sites. PMs are generated in a wide range of locations; therefore, they must be managed in real time at various locations within construction sites for practical management of the PMs. However, no such systems exist currently. Therefore, this study aims to develop a system that can manage PMs in real time at multiple locations in a construction site using the Internet of Things technology. Accordingly, measuring instrument, network, and program services were developed as system components, while considering the characteristics of construction sites, and the construction site PM monitoring system was developed by integrating these components. Finally, performance certification and field application tests were performed to verify the developed system. The construction site PM monitoring system (CPMS) achieved grade 1 for reproducibility, relative precision, and data acquisition rate, and grade 2 for accuracy and coefficient of determination. Thus, it received a performance certification of grade 2, in total. In particular, regarding accuracy, which is a shortcoming of the light-scattering method and represents the accuracy of measurements, the CPMS was found to have an accuracy of 74.2%.

## 1. Introduction

With increasing industrialization and urbanization, air pollution has emerged as a global issue, and the dust generated from construction processes is a major social problem that must be solved [1,2,3]. This dust, generated on construction sites, poses serious risks to the health of field workers and residents living near construction sites [4]. The World Health Organization (WHO) has established guidelines on the annual and daily average concentrations of particulate matters (PMs), and based on these guidelines, each country is expected to establish environmental standards according to their respective situation [5]. While many countries use these WHO guidelines as the basis for their PM concentration management policies, other policies to regulate PM emission behaviors when the PM generation exceeds the set threshold are also being actively enforced [6,7,8,9]. One such policy that is currently enforced in South Korea limits the PM concentration on construction sites, which is a management objective under the emergency measures for reducing ultra-fine dust in air [10]. This policy takes effect when the PM2.5 concentration exceeds or is expected to exceed the threshold on the same day or the next day, and upon enforcement, it is mandatory or recommended to shorten construction work hours. However, this policy is applicable only when the general PM concentration in the atmosphere is high, and it is unrelated to the PM concentration on construction sites. Hence, it has a limitation in comparison to actual PM management on construction sites. Moreover, representative national PM control systems such as Winter Emergency Measures in Italy; Fine Dust Alarm in Germany; and South Coast Air Quality Management District, Bay Area Air Quality Management District, and National Ambient Air Quality Standards (NAAQS) in the US also focus on the measurement of PM concentration of a broad area [11,12,13,14,15,16]. However, it is inappropriate to apply the systems to evaluate detailed PM generation in small scale areas such as construction sites, since the measurement points of these control systems are spread widely, and the measurement points are spaced apart. Hence, it is necessary to develop a PM monitoring system specialized for construction sites to manage the PM generation events precisely.

Because construction sites have a large scope and dust-generating activities occur at several locations, PMs must be managed organically at multiple locations instead of at a single location within the site, for practical PM management. Currently, the concept of the Internet of Things (IoT) is being used in many studies for organic air pollution management [17,18,19]. The term “IoT” was first used in 1999 by Kevin Ashton, and the conceptual definitions of this term vary widely. Some studies interpreted IoT from the perspective of services, such as providing new services (things and people) to end users by automatically collecting information through integrated technology with smart devices (things) [20,21]. Others interpreted IoT as a technology, either from the perspective of an ecosystem among things (pure technical perspective) or from the perspective of an ecosystem of things and people (socio-technical perspective) [22]. Such differences in perspective originate from the presence or absence of services provided to end users, but the common requirement among them is that the “things” should recognize the general context and detect and respond to changes based on organic communication between things or between things and people [23].

The goal of this study is to develop an IoT-based PM monitoring system that can perform organic field management to protect field workers and residents living near construction sites by adopting the IoT concept from a service perspective, which can provide site managers with real-time services. In particular, measuring instruments, networks, and program services comprising the system were developed in accordance with the construction site environment, and a construction site PM monitoring system (CPMS) was built by integrating them. Finally, the developed system was verified through performance certification and field application tests.

## 2. Background

### 2.1. PM Sensor

The essence of a fine-dust measuring instrument is the actual measurement method used, and this measurement method should be considered first when choosing a PM sensor. The different types of measurement methods are the weight concentration method, the beta-ray method, and the light-scattering method. Each measurement method has its own unique characteristics and is selected depending on the application. Among them, the beta-ray method and the weight concentration method are official fine-dust measurement methods specified in the Air Pollution Process Test Standard of South Korea, and owing to their high accuracy, their measurements are used as the standard measurement values [24]. In contrast, the light-scattering method, which irradiates fine particles and measures the concentration of particles using the scattered light, has a higher error rate than the beta-ray method and weight concentration method. However, its advantage is that data can be obtained more simply with it because the sensor is small and inexpensive, and a separate dust collection process is not necessary [25]. Consequently, domestic studies related to construction site PMs have adopted the light-scattering method as a measurement method to respond to changes in various situations in the field, such as construction sites, or to perform temporary measurements for a certain period [25,26,27]. In particular, the criteria for judging the suitability of a measurement method for construction sites include real-time data output, mobility, economy, and accuracy. After reviewing the suitability of each measurement method by considering these factors as in Table 1, the light-scattering method was found to be the most suitable [27]. However, the accuracy of acquired data should be verified when using the light-scattering method. 

Since 2019, South Korea has enforced a simple fine-dust meter performance certification system for institutions designated by the President of the National Institute of Environmental Research [28]. This system evaluates five performance items (reproducibility, relative precision, data acquisition rate, accuracy, and coefficient of determination) and assigns one of four grades (grade 1, 2, 3, off-grade) to each meter [29]. Herein, simple fine-dust meters generally refer to portable fine-dust measuring instruments that use the light-scattering method. The performance of such simple fine-dust meters was compared to the measurement performance of the weight and beta-ray methods, which are proven to be reliable [29,30]. This was to compare the measurement performance of the light-scattering method against the weight and beta-ray methods. In particular, the data accuracy, which is a shortcoming of the light-scattering method, can be verified by evaluating the accuracy of the evaluated items. In general, a measurement is considered to be reliable if the level of accuracy is of grade 1 (accuracy higher than 80%) or grade 2 (accuracy higher than 70%). Therefore, it is necessary to achieve a grade 2 or higher accuracy of the measuring instrument for the light-scattering method.

### 2.2. IoT-Based PM Monitoring System

An IoT-based PM monitoring system involves sending the data measured using a measuring instrument through the network and providing services to users. Therefore, the system should be examined in terms of measuring instruments, networks, and services, while considering the characteristics of the system construction target. Construction sites are characterized by an extremely wide monitoring range, and the occurrence of dust-generating activities at several locations. Consequently, the system must be set up such that the field managers can check the dust-generating locations and take measures in real time. Furthermore, because the major dust-generating locations change over time and workers are exposed to harsh outdoor environments as construction progresses, the system must be easy to install at various locations and possess sufficient durability. To include such construction site monitoring characteristics in the system development, this study examined mobility (miniaturization, portability) and outdoor durability of the measuring instrument, and also examined connectivity and communication range of the network at existing works. In terms of services, the types of data access to end users were examined, as shown in Table 2. 

Kang et al. developed a real-time environmental pollutant monitoring system called MONVID, which contained a PM sensor of the light-scattering method for application to construction sites [31]. MONVID adopted the recommended standard 485 (RS485), which allows a wide communication range at low cost, as the communication method, which provides data services through the internet and mobile. RS485 is appropriate for construction sites because it not only supports communication over a wide range, but it is also less affected by obstacles. Moreover, the data service method of MONVID also has the advantage that it is easy to manage the site because the site manager can check real-time data anytime and from anywhere. 

Smaoui et al. adopted TSI DustTrack II, which can perform mobile measurements, while developing a dust-sensing technology for construction sites using location tracking technology [32]. It is a small measuring instrument that can perform real-time mobile measurements and has assured portability through the use of batteries. However, such portable products have a limit to continuous measurements at a construction site, and their durability should be considered in relation to a continuous power supply and long-term installation.

In addition, most studies on the development of PM monitoring systems focus on the construction of systems for an indoor environment [33,34,35]. Because the measuring instrument of indoor PM monitoring systems was not exposed to snow and rain in outdoor environments, outdoor durability has not been concretely tested, but they were observed to be structurally advantageous for miniaturization. Moreover, in terms of network and services, these instruments are characterized by the provision of web-based data services using the Wi-Fi protocol, which is advantageous for applications in indoor environments. Such technologies can be used to provide indoor monitoring services to management offices on construction sites or in affected areas near construction sites.

### 2.3. Framework of System Development

**Figure 1:**
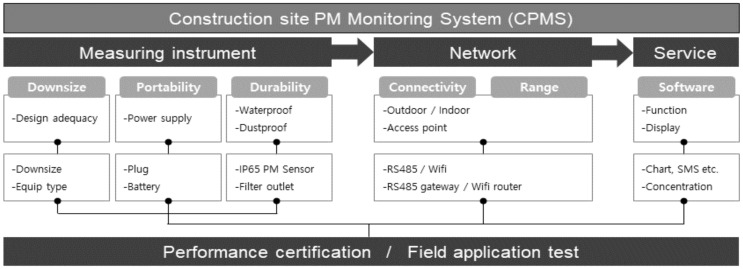
Framework of CPMS development.

To develop the IoT-based CPMS, this study divided the system components into measuring instruments, networks, and services. Subsequently, the development results were derived for each component. Finally, the measurement performance and normal operation were verified through performance certification and field application tests of the total system. The framework of this study is illustrated in Figure 1.

## 3. Materials and Methods

### 3.1. Measuring Instrument Development

The measuring instrument developed in this study was miniaturized and its weight was reduced to make it portable for its mobility. Therefore, the volume and weight of the sensor itself were reduced by adopting a low-cost PM sensor, and a compact module was produced by adopting temperature, humidity, and CO_2_ sensors. Furthermore, the mounting joint of the measuring instrument was designed in line with the construction site post-bar specification, having an inner diameter of 48 mm, so that it was easily mounted on a stand-type on-site post-bar. However, because it can be difficult to set up a post-bar when installed on construction equipment or at a site office, the exterior shape of the measuring instrument was developed so that it could be installed on a wall or floor using an attachable cradle.

**Figure 2:**
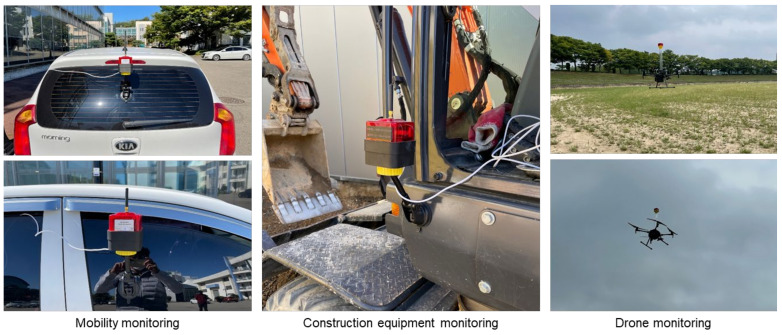
Mobile installation of CPMS.

Supplying power to the measuring instrument in fixed and mobile monitoring situations has to be easy. Thus, to enable one measuring instrument to be flexibly replaced with a plug-type or a battery-type depending on the situation, the rated power was set to 5 V/2 A and a USB-type power connector was selected. In particular, it was verified that when the battery-type was used, the instrument worked continuously for up to approximately 1.5 days with a low-capacity battery of 10,000 mA h and for up to approximately 10 days with a high-capacity battery of 60,000 mA h. Therefore, it was considered that power could be supplied easily using a low-capacity battery to improve portability for short-term monitoring of approximately one day, and by replacing it weekly with a high-capacity battery at locations requiring constant monitoring, where it is difficult to install an electric plug. The examples of installing the developed measuring instrument for mobile monitoring are shown in Figure 2.

In addition, outdoor durability was assessed for the development of the measuring instrument. Because of the nature of construction sites, the measuring instrument is likely to be exposed to bad weather conditions such as snow and rain, and a high-concentration PM environment, which can lower the durability of the measuring instrument and cause difficulty in maintenance. To enable protection from such risks, a PM sensor with dustproof and waterproof grades should be considered, and outlets that can protect sensors from external conditions may be used to enhance durability. First, the PM sensor with dustproof and waterproof grades itself can be reviewed based on the IP grade, defined in accordance with the international standard IEC 60529 [36]. Hence, this study adopted cubic PM2009 as the PM sensor of IP65 grade in the measuring instrument.

**Figure 3:**
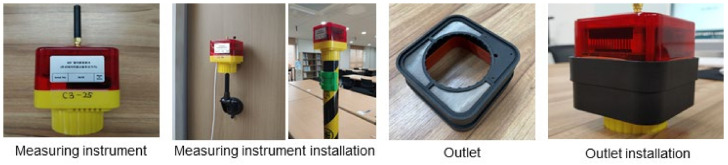
Measuring instrument of CPMS.

Furthermore, this study developed a measuring instrument protection outlet to enhance the durability of the measuring instrument in harsh environments on construction sites. Therefore, an appropriate outlet structure and material were examined in terms of waterproof performance and the ease of maintenance of the outlet. First, an appropriate outlet in a shape that covers the dust collector to protect the module from rainwater was designed for improving the waterproof performance, and a waterproof packing was applied to the joint of the outlet and measuring instrument. Among them, PA12 was selected as the material for the dust collector cover to improve the weight reduction and durability of the outlet. In addition, silicon was selected as the packing material for enhancing the ambient heat resistance, so that the packing effect could be maintained at high temperatures in summer. In terms of dustproof performance, a mesh filter, through which fine particles smaller than 10 μm can pass, was used to filter out large particles of scattering dust from construction sites, while having negligible effect on the PM measurements. The size of the pores was determined according to the mesh size. In this study, the effect on PM measurements was analyzed for 150-, 250-, and 400-meshes, corresponding to the scattering dust diameter (100 μm), Korean total suspended particle (TSP) diameter standard (50 μm), and the TSP diameter standard of the US and Europe (30 μm). The results showed that the measurements decreased significantly when meshes of 250 and 400 were compared to the no-mesh filter. In the case of the 150-mesh, the decrease in the measurement was relatively small. This implies that for 150-mesh, particles larger than 100 μm were filtered, while fine particles smaller than 10 μm, the measurement target, easily passed through the mesh filter. Therefore, this study selected the 150-mesh as the filter for waterproof and dustproof outlets. For the mesh filter material, stainless-steel was selected so that the filter could be reused after washing, semi-permanently for easy maintenance of the outlet. The CPMS developed in this study is shown in Figure 3. 

### 3.2. Network Development

**Figure 4:**
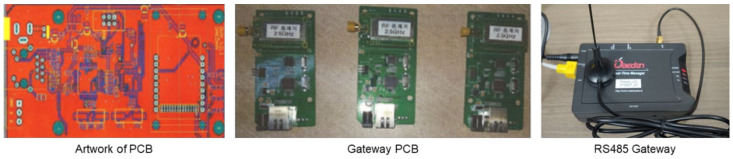
RS485 gateway for CPMS.

In this study, the communication range was examined to set up an IoT network, according to the structure and size of the construction site, while selecting the communication method for measuring instrument data. The standard communication methods for IoT monitoring systems generally include Zigbee, Bluetooth, and Wi-Fi. Among them, Zigbee and Wi-Fi achieved the appropriate communication range for construction sites. However, owing to the nature of construction sites, severe communication interferences were caused by construction equipment and structures. Thus, these communication methods were considered inappropriate for remote communication on construction sites. Finally, this study adopted RS485, which allows long-distance communication and has less communication interference from obstacles, as a remote communication method. For the short-distance communication method, Wi-Fi, which can use a router generally used indoors without a separate hub, was selected, making it convenient to apply the measuring instrument in the field office or indoor spaces near the site. Although the RS485, which was selected as the remote communication method, cannot be used for short-distance communication, it can be used together with Wi-Fi for indoor monitoring. The results of reviewing the communication methods for building the construction site network are summarized in Table 3. The RS485 gateway for configuring the CPMS network access point was developed, as shown in Figure 4. 

### 3.3. Software Development

Regarding the PM monitoring technology software, the user functions must be reviewed together with the implementation method using a display. Because of the nature of construction sites, construction workers, who are classified as outdoor workers, require health protection measures because they are exposed to PMs for a long time. Residents in downtown areas close to construction sites can also be affected by PMs. Consequently, the main users of the CPMS software developed in this study were specified as field workers, who were directly affected by the on-site PMs and must also control damage from PMs to nearby areas. Field workers must also be able to simultaneously check emission events that occur at multiple locations on the construction site and intuitively manage the trend of environmental data. In addition, they must be able to recognize and respond to high-concentration situations at specific locations in real time. Therefore, this study developed a web-based construction site PM monitoring program customized for field workers equipped with simultaneous monitoring of data for all sensor points, hourly data charts, hourly data storage and output, and high-concentration short message service (SMS) notification functions. The “Home” page allows users to simultaneously see real-time data from all measuring instruments, and check the operation status of the measuring instruments. The “Chart” page provides graphs to allow users to intuitively see changes in data for a certain period by selecting the data of the desired measuring instrument as necessary. The “Report” page can be used to print the data journal of the desired date or output data of one or five-minute units for the desired period as an XLS file. The “My Product” page supports efficient network management by registering an access point (gateway) as a product, and registering devices interconnected around the registered product. The “Manual” page allows users to download registered data, including various manuals, using links. Lastly, the SMS page, which is the most important component of this program, can be used to register the SMS receiver list and send notifications for events to these receivers when events fulfilling specific conditions occurred continuously for a specific period in the specified measuring instruments. This allows users to immediately respond to and manage events by recognizing the locations and situations of the events in real time. On the SMS page, not only the PM, but also temperature, humidity, and CO_2_ conditions can be set so that SMS services can be provided for integrated environments.

Existing PM monitoring systems use concentration and index methods to show the results on the display of the software. The concentration display method shows, on the display, the actual data measured by the sensors. The index method converts the data into an index and displays that index on the display. Representative examples of the index method are the air quality index of the U.S. EPA and the comprehensive air-quality index of South Korea. The concentration display method is suitable for situations such as emergency high-concentration reduction measures using a reference value for the concentration, whereas the index method is advantageous for users to make intuitive judgments about the health risk from the situation. In this study, the concentration display method was adopted by default so that the field workers, who were the main users of the CPMS software, could manage the PM concentration with quantitative values. In the future, if the range of service users is expanded, the index method can be used in conjunction with it. 

**Figure 5:**
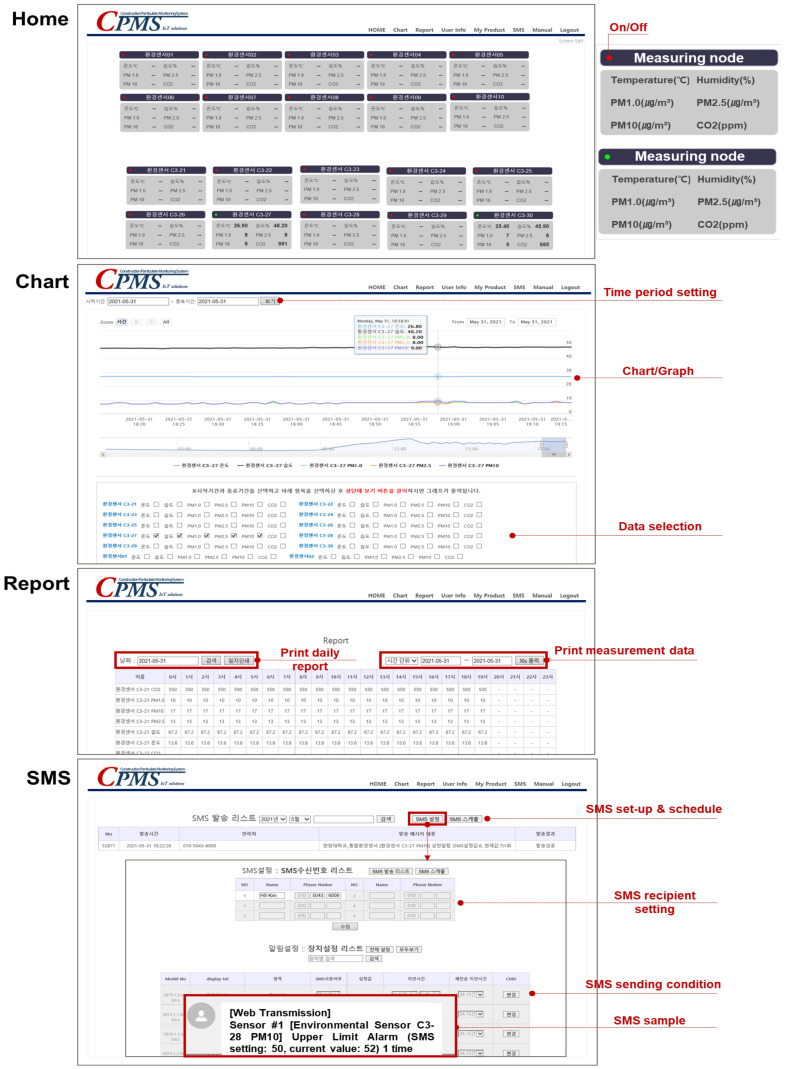
Main screens of the CPMS software.

The main screens of the CPMS software developed in this study are illustrated in Figure 5. This software was developed to facilitate real-time use on the web browser of portable devices, as well as computers.

### 3.4. Performance Certification and Field Application Test

**Figure 6:**
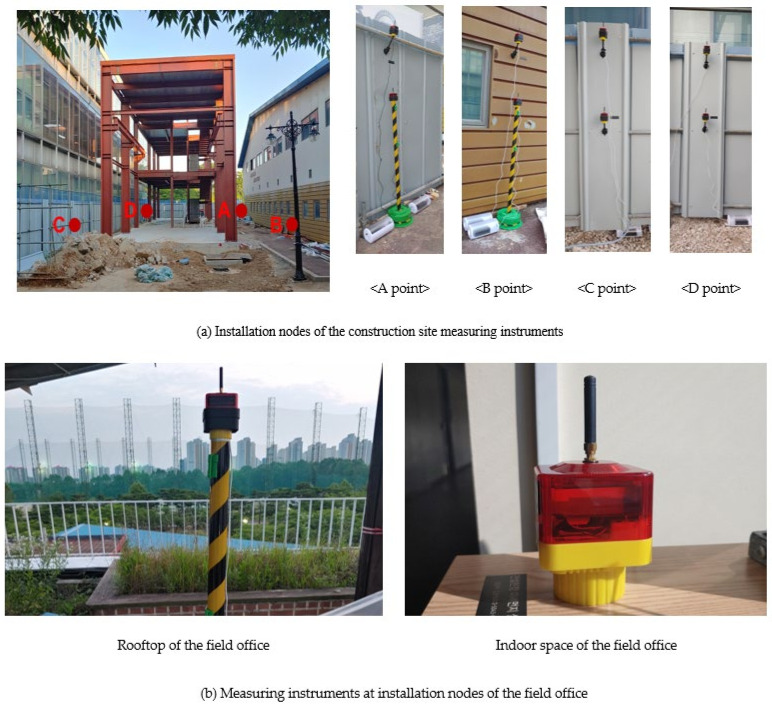
Test site network setup.

To verify the measurement performance of the CPMS developed in this study, grade certification was performed for reproducibility, relative precision, data acquisition rate, accuracy, and coefficient of determination obtained through a simple fine-dust meter performance certification system. In addition, tests were performed to verify the normal operation and durability of the network by applying the CPMS to a construction site. Because there was no separate field office at the construction site, a neighboring building was assumed as the field office, and the network was configured by setting the access point there; the measuring instrument nodes were installed at four points in total at the boundary of the construction site. In addition, the nodes were installed at a point inside the field office and on the rooftop of the field office. The test monitoring network was observed for two months from 22 June to 21 August 2021. The node installation points for the network configuration of the test site are shown in Figure 6.

## 4. Results

### 4.1. CPMS Composition

**Figure 7:**
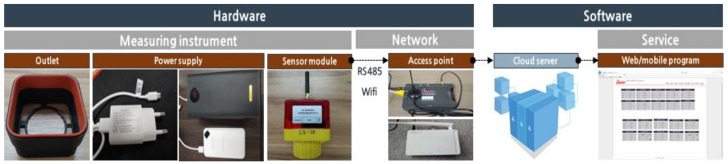
CPMS network composition.

The basic structure of the CPMS developed in this study largely consisted of hardware and software sections. The hardware section included all the components of the network for transmitting data from the sensor nodes to the server, whereas the software section indicated the process of creating new services through this network. The detailed composition of the system developed in this study is shown in Figure 7.

### 4.2. CPMS Performance and Field Test Results

From the simple fine-dust meter performance certification test, which was performed to verify the measurement performance of the CPMS, the CPMS received grade 1 for reproducibility, relative precision, and data acquisition rate, and grade 2 for accuracy and coefficient of determination as in Table 4. Thus, it received a performance certification of grade 2, in total. In particular, regarding accuracy, which is a shortcoming of the light-scattering method and represents the accuracy of measurements, the CPMS was found to have an accuracy of 74.2%. 

**Figure 8:**
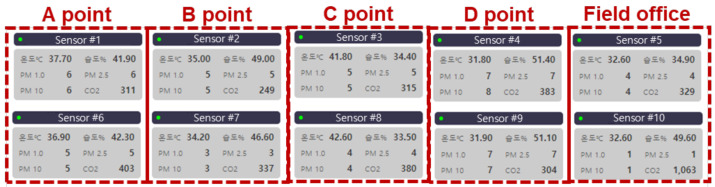
Real-time data services of the program in field test.

In addition, to verify the normal operation of the developed CPMS, tests were conducted by installing two nodes, each at four points, on an actual construction site. The results confirmed that the data collected through the network were displayed on the Home page of the CPMS software, as shown in Figure 8. Additionally, all the multisensory nodes installed outdoors worked normally in the hot, humid, and windy summer environment of South Korea. 

**Figure 9:**
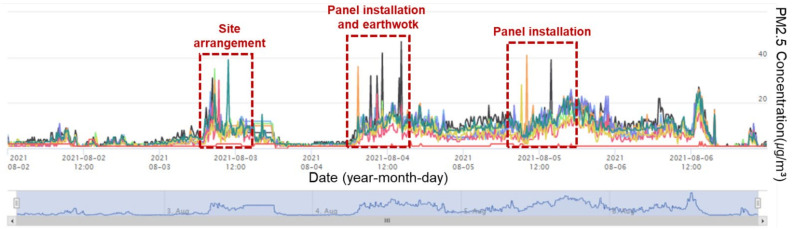
PM2.5 concentration chart (2 August~6 August).

**Figure 10:**
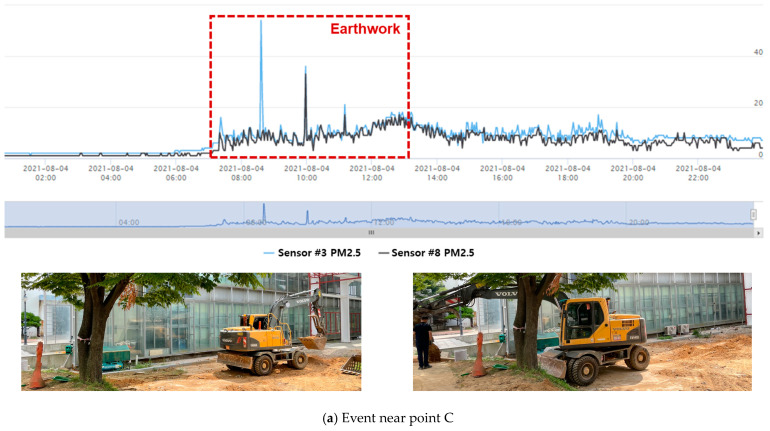

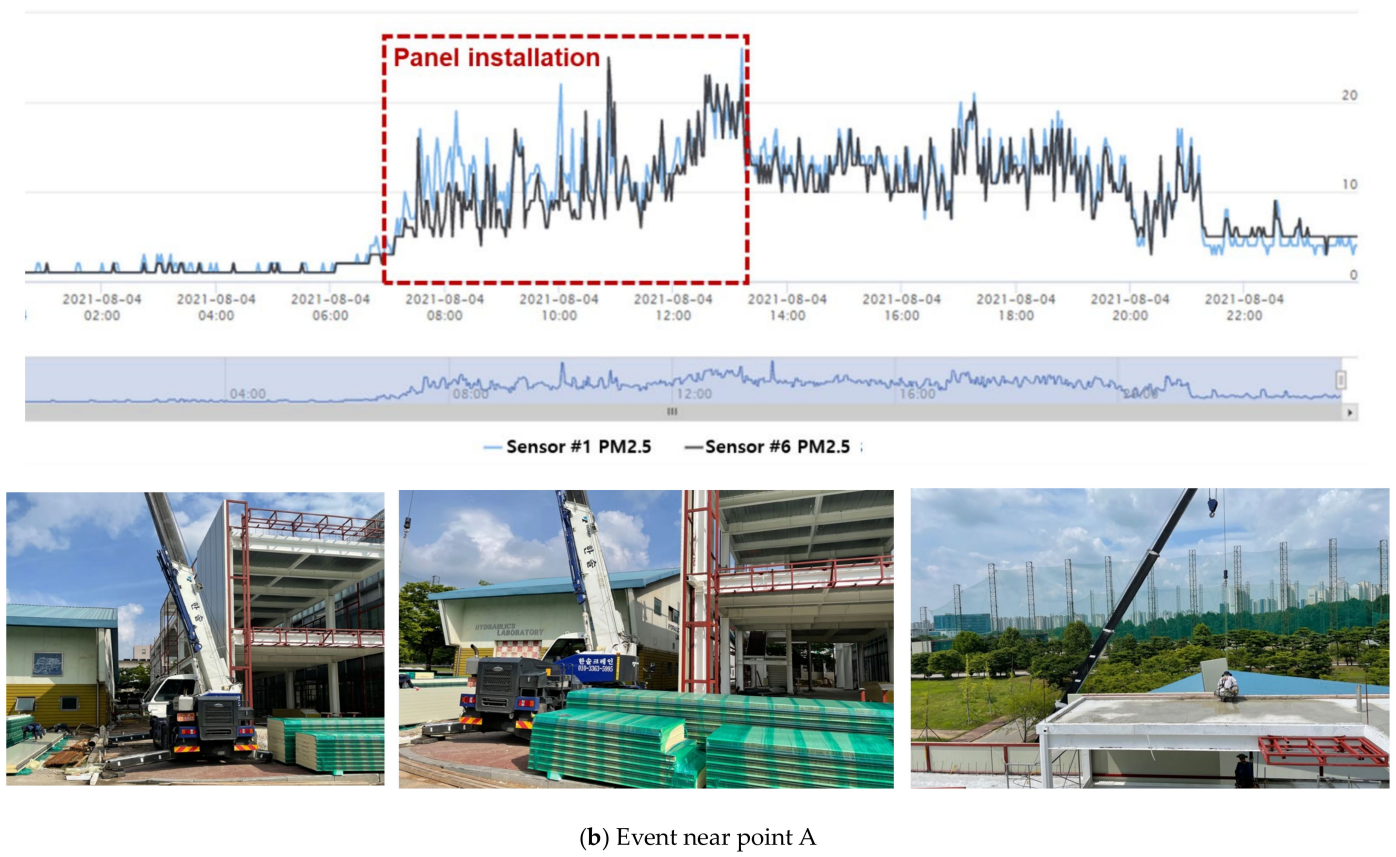
Dust generating events (4 August).

When the PM concentration in the air was high or construction activities that produced PMs were performed at the test site, a distinct change in concentration was observed, as shown in Figure 9. In particular, when construction activities that produced PMs were performed at specific locations, the changes in the measured concentration data were observed through the measuring instruments installed near the corresponding locations, as shown in Figure 10. This indicated that efficient management is possible for PMs generated at specific locations on the construction site by site managers using the CPMS. Furthermore, the SMS function of CPMS software can be used to support field management more efficiently. In this field test, a notification concentration was set for the sensors installed at the site, and the concentration at the location where a high concentration of PM exceeding the set reference value occurred was recorded in real time. This confirmed that it is possible to immediately manage construction activities that produce PMs.

## 5. Conclusions

In this study, an IoT-based PM management system that enables organic construction site management was developed, so that field workers and residents around construction sites can be protected from the PMs generated on construction sites. The conclusions of this study are as follows.

1. The aspects of measuring instruments, networks, and services were examined, while considering the characteristics of construction sites where the system was to be installed. Mobility and outdoor durability of the measuring instrument were examined. Regarding the network, connectivity and communication range were examined, while regarding services, the developed forms of data access software for end users were examined.
2. The PM measuring instrument was developed to allow miniaturization for mobility and facilitate field installation, so that electric plugs and batteries could be used for power supply. The outdoor durability was enhanced by adopting an IP65-grade PM sensor and developing a dustproof and waterproof outlet.
3. For the system network, RS485, which exhibits negligible jamming by obstacles and long-distance communication, was adopted as the main communication method, while considering the structure and size of construction sites. In addition, the Wi-Fi method, which can use the generally indoor routers, was adopted for easy application of measuring instruments in field offices or indoor spaces near construction sites.
4. For the software services of the system, the concentration display method was adopted as the default display method, instead of the index method. This was undertaken so that the PM concentration could be managed precisely using quantitative values, along with the major functions of chart, report, and SMS features for the convenience of the construction site managers, who were the main users.
5. Finally, the CPMS was configured with hardware (measuring instruments and networks) and software services. A simple fine-dust meter performance certification test was performed to verify the measurement performance of the CPMS, and an overall performance certification of grade 2 was obtained. The field application tests showed that the system worked stably in harsh construction site environments and could support real-time site PM management by notifying the site managers, and by measuring high-concentration PMs occurring at and around each node.
6. The CPMS is practical, in that it is a technology that supports PM management by construction site managers based on their location. In the future, it is expected to be developed into a system that can directly control PMs on construction sites by applying various PM control technologies and going beyond the concept of supporting site management.

However, long-term field tests are required for this system, and the overall stability of the system must be enhanced by investigating mid-to long-term maintenance methods for the system. In addition, the South Korean government has recently constructed the Public Safety-Long Term Evolution (PS-LTE) network to improve the efficiency of disaster response services, and air pollution management by government agencies through the PS-LTE is also being discussed [37]. The CPMS network developed in this study was configured to facilitate the management of single construction sites. However, to support comprehensive PM management for construction sites at the central or local government level, methods to connect it with the national communication network in each country, such as the PS-LTE in South Korea, should be studied. Moreover, it is necessary to connect the local air monitoring systems to CPMS to monitor the wind direction and wind speed, which influence the flow of particulate matter emissions. Wind is a crucial factor for determining whether a given increase in pollutant concentration comes from construction activities or is the result of transporting pollutants from the environment [38]. Therefore, additionally, the influence of wind should be considered in future system improvement.

## Figures and Tables

**Table 1:** Suitability of measurement method according to the characteristics of construction site.

Items	Construction Site PM Measurement Method		
	Weight Concentration Method	Beta-Ray Method	Light-Scattering Method
Real-time data output	X	X	O
Mobility	X	X	O
Economy	High equipment and analysis costs	High equipment cost	Low cost
Accuracy	High	High	Low

**Table 2:** Studies on IoT-based PM monitoring systems.

Studies	Measuring Instrument			Network		Service
	Mobility		Outdoor Durability	Connectivity	Long Range	Data Access Software
	Downsize	Portability				
[31]	X	X	X	RS485	O	Web, Mobile
[32]	O	O	X	-	X	Program
[33]	O	X	X	Wi-Fi	X	Web
[34,35]	O	X	X	Wi-Fi	X	Web

**Table 3:** Selection of communication method for the construction site PM measuring instrument.

Classification	Protocol			
	Zigbee	Bluetooth	Wi-Fi	RS485
**Range**	10–100 m	10 m	20–100 m	Up to 1200 m
**Main items**	-Remote distance: Large interferences from obstacles -Short distance: A separate hub is necessary	-	-Remote distance: Large interferences from obstacles -Short distance: A router is used	-Remote distance: Small interferences from obstacles -Short distance: A separate hub is necessary
**Selection**	X	X	O (short distance)	O (remote and short distances)

**Table 4:** Certified performance grade of CPMS.

Performance Categories	Range	Grade
**Reproducibility**	80% <	1
**Relative precision**	80% <	1
**Data acquisition rate**	80% <	1
**Accuracy**	70% <, and ≦ 80%	2
**Coefficient of determination**	0.7 <, and ≦ 0.8	2
**Overall**	-	2

## Data Availability

Not applicable.
